# Erysipelas

**Published:** 1848-06

**Authors:** Bransby B. Cooper


*Erysipelas.—From Lectures on Surgery in the London Medical Gazette.* By Bransby B. Cooper, F. R. S., &c.—You may consider this subject, gentlemen, as belonging rather to the province of the physician than to the surgeon; but erysipelas so frequently follows local injury, that, unless a surgeon is acquainted with the phenomena connected with this disease, and the appropriate treatment for their relief, he would constantly be obliged to transfer the care of his patients to the hands of the physician. In fact, no better instance than erysipelas can be adduced to prove the necessity for a surgeon to render himself thoroughly acquainted with loco-constitutional diseases.

Erysipelas is an inflammation of a very peculiar character, attacking the external surface of the body, and indicating all the usual signs of a morbidly increased action, attended with redness, heat, swelling, and pain, each of these offering characteristic marks. Sometimes it seems to attack the skin only, unattended with any concomitant constitutional disturbance: it is then termed erythema.

The redness of erysipelas is remarkable, on account of its sudden disappearance upon the slightest pressure, leaving a white spot; but the redness almost instantaneously returns upon the removal of the force. The intensity of the colour varies very much in different cases, and this variety depends more upon the constitution of the patient than upon the severity, or any peculiarity in the disease itself.

The heat of the affected part is of a burning character, and is described by the patient as producing a dull pricking, or rather tingling, sensation. The degree of swelling depends upon the circumstance of the sub-cutaneous tissues being affected or otherwise; for, when the skin alone is inflamed, there is little or no swelling or tension, and, in fact, the inflammation is at this period to be considered as merely erythematous; but, immediately upon the implication of the cellular membrane, swelling becomes a prominent feature of this disease.

The pain is seldom acute, but is said to resemble a tingling stiffness, and it produces invariably a restlessness which is highly characteristic of the disease. If pressure be applied to the inflamed part, the pain and uneasiness are very considerably increased. The local symptoms are generally preceded by considerable constitutional disturbance—such as pain in the head, full pulse, loss of appetite, rigors followed by dejection, debility, sometimes vomiting, and early delirium, if the head be the seat of the disease. Although these symptoms likewise frequently attend common pyrexia, there is something so peculiar in their nature—so sudden in their development—that every experienced nurse in an hospital recognises them as premonitory signs of erysipelas.

Medical writers have distinguished erysipelas by the terms phlegmonous, bilious, and local or erythematous. Were I to take this detailed view of the disease, I admit, gentlemen, that I should be rather encroaching upon the province of the physician. I shall therefore dwell especially on the phenomena resulting from local injury—"traumatic erysipelas."

The question naturally arises, whether injury to any tissue can in itself produce the specific action of erysipelas without accessory constitutional predisposition. I am myself inclined to reply in the negative; for I believe that this disease is the result of a constitutional derangement, arising chiefly either from epidemic or endemic causes, for how frequently is it observed in this and every other hospital, that, when one patient has become affected with erysipelas, others are found liable to its attacks from causes much too slight to be considered capable of producing the like result under ordinary circumstances. This is so well known, that every hospital surgeon postpones the performance of surgical operations even after the patient has been prepared for the ordeal, if he is aware that erysipelas is present in the ward.

It is quite true that a healthy person would probably resist the infection; but, under the depressing influence inseparable from an operation, it would be incurring an unwarrantable risk to expose a patient to the continued influence of such a poison, particularly if the case is one which will, under any circumstances, admit of delay.

There is certainly a peculiarity in traumatic erysipelas, with respect to its so frequently following wounds of the head and face; and I consider that this may depend upon the insertion of all the muscles of this region into the skin, the tissue invariably first affected by this peculiar description of inflammation.

Hence, in the case of persons suffering from an attack of erysipelas in the face, the most complete state of quietude, and absence of all mental excitement, are desirable, as affording the only means of preserving these muscles in a perfect state of rest, as they are immediately put into motion by the operation of almost every external circumstance, or by the least mental disturbance.

Another peculiarity in erysipelas, not yet alluded to, is its erratic tendency, or what is technically termed "metastasis," which constitutes one of the most remarkable features of this complaint.

The consideration of this fact forms a very important point in regulating our practice, and especially in erysipelas of the head; for, however proper it may be to attempt suddenly to subdue erysipelatous inflammation of the limbs or trunk, by the application of evaporating lotions, or any other means of abstracting the abnormal heat of the affected part, such treatment is quite inadmissible in erysipelas of the head or face, owing to the danger of producing metastasis to the membranes of the brain.

I have more than once seen a patient delirious a few hours after cold had been applied to an erysipelatous scalp, and restored as quickly to consciousness by the substitution of warm fomentations for the evaporating lotion. The rationale of this is sufficiently obvious: the action is due to the free anastomosis between the vessels of the pericranium and of the dura mater, through the substance of the bones of the skull; so that any cause that propels the blood from the pericranium must produce a proportionable influx into the vessels of the dura mater.

Patients attacked by erysipelas (more especially in this metropolis) bear depletion very badly, and there are but few cases in which general blood-letting can, in my opinion, be admissible.

Leeches should never be employed in erysipelas, as their bite becomes a fresh source of irritation; and, indeed, it is frequently the exciting cause of this peculiar character of inflammation.

The only antiphlogistic plan, therefore, left, is that of acting upon the secretions, which effect is readily produced by employing the following remedies:—℞ Hyd. Chloridi, gr. iss.; Pulv. Jacobi veri. gr. iij. M. ft. Pilul.; Magnes. Carbonat. gr. x. ℞ Sodæ Sesquicarbonat. ℈j.; Vin. Ipecac. ʒss; Mist. Camphoræ, ℥j. M. ft. Haustus adde Succi Limonis Recentis, ℥ss. et in statu effervescentia sumendus bis terve quotidie. Should the patient evince any typhoid symptoms, ammonia should be substituted for the soda.

If there be much tension of the skin, attended with small blisters, without remission of febrile symptoms, it should be punctured in several places, to allow of transudation of the effused serum. This operation generally affords great relief. With respect to the long incisions recommended by some surgeons, I consider that practice to be worse than useless, unless there be extensive sloughing of the cellular membrane, which will very rarely occur if punctures be made as soon as the necessity for such relief is indicated by the tension of the skin; indeed, I have known fatal sloughing sores induced by the practice of incisions, and in more than one case death occurred from the hæmorrhage immediately resulting from the operation.

When erysipelas becomes diffused, the vivid discoloration of the skin diminished, the tongue dry, and the general signs of debility manifested, stimuli are required; but in common cases generous support is preferable to stimulus: I therefore usually prefer porter to wine or brandy, excepting under the circumstances above mentioned.

Where the inflammation of erysipelas has a great tendency to spread, it has been recommended to attempt to check its course by cauterising with lunar caustic the skin above the inflammation. Some have recommended mercurial ointment to be employed with the same view; and indeed I have seen both of them produce beneficial results by circumscribing the extent of the inflammation. I presume that the lunar caustic and the mercurial ointment close the pores of the skin wherever it is applied, and, preventing the natural cutaneous exhalations, set up a new action, and so tend to prevent the spreading of the erythematous inflammation; for, as far as I have observed, any other ointment will answer the purpose as well as the mercurial.

This fact would certainly lead one to the belief that erysipelas is, at any rate at its commencement, a cutaneous disease, and the extension to the subcutaneous tissues the result of a secondary action.

Vesicles generally form in those cases which do not terminate by resolution; hence erysipelas has been classed under the order Bullæ, by Dr Bateman.

In debilitated constitutions, diffused abscesses frequently follow erysipelatous attacks, sometimes even at a distance from the originally inflamed part. Indeed, I have occasionally seen abscesses follow wounds around which no erysipelatous inflammation had occurred, and yet subsequently diffused cellular membranous abscesses have formed in different parts of the body, attended with considerable local inflammation; but whether these could be regarded as erysipelatous affections, I have frequently had much difficulty in determining. What I mean to express is, gentlemen, that it is often very difficult to distinguish the inflammation resulting from the formation of abscess in debilitated patients from phlegmonous erysipelas. In these cases, also, as in erysipelas, the abscesses are rarely limited by an adhesive boundary, but are diffused, indicating the extreme debility of the patient.

When abscesses result from erysipelas, they rarely extend beyond the subcutaneous cellular membrane, and do not appear to lead to absorbent inflammation, probably in consequence of the freedom with which the matter becomes diffused; while, on the contrary, when pus is formed in more deeply seated structures, as in subfascial and thæcal abscess, it is pent up by the inextensible tissues, and leads, therefore, to more urgent constitutional disturbance, and requires early provision for its evacuation.

Great care and attention are required after a patient may have apparently recovered from an attack of erysipelas, owing to the great tendency to relapse which generally exists in such cases; and it may, perhaps, be said—at least so my experience leads me to believe—that a person once attacked by this disease is ever after liable to its return from any exciting cause to inflammation—a circumstance which would seem to prove that the disease depends more upon peculiarity of constitution than upon the nature of the accidental injury, or even, perhaps, than upon any epidemic influence.

I have said, gentlemen, that it might be considered a deviation from my province to speak of bilious erysipelas, and other particular constitutional derangements modifying this disease; still, do not for one moment imagine that I consider it unnecessary for you to study, and *scrutinously* too, the peculiarities, diathesis, and temperament of your patient; for you must remember that the slightest local injury can never occur without the restorative process being influenced by the age, sex, habit, and constitution of the subject; and whoever fancies that, because he has made himself acquainted with the name of the disease, he can at once apply some well known appropriate remedy, will never advance beyond empiricism, nor establish his title to be considered in the light of a scientific practitioner; and I would almost say that his practice would be dangerous in proportion to his rapid decision in the classification of disease, if that alone be his aim. After what has been said, as to the tendency to erysipelas following the wounds of the scalp, and skin of the face, let me urge you, gentlemen, to be cautious how you undertake even trivial operations, on these regions of the body, without first having duly prepared your patient for the effects they invariably produce in the system. In some cases you may be requested to remove small encysted tumors from the scalp—an operation so trivial that it may be executed by a mere tyro in the profession—but even the most experienced and skilful surgeon may risk the life of a patient, and his own reputation, by want of a little precaution.

Never, I say, undertake such a task without first well ascertaining the actual state of your patient's health, as to the absence of any organic disease, the condition of the bowels, state of the urine, and natural performance of the functions essential to a healthy state of body.

Several years ago I removed an encysted tumor from the head of a patient. Upon making a mere incision through the skin it immediately turned out, the operation of extracting it not occupying more than a minute. On the third day I considered my patient convalescent; on the fourth I was suddenly sent for to see him, and found that a most startling change had taken place in his condition. I should not have recognised him; his head was swollen to twice its natural size; not a feature could be discerned; and his complaints were urged in muttering delirium. I immediately ordered him (as his bowels were costive) a large dose of calomel, fomented his head and face, punctured the scalp, and prescribed diaphoretic effervescing draughts. The day following he had but slightly improved, although his bowels had been freely opened, and I immediately proposed a consultation. The gentleman who met me recommended bleeding—a remedy to which he especially trusted in all cases of febrile action. But as the patient had a very dry tongue, attended with delirium, and was complaining of great thirst, muttering in almost inarticulate sounds his desire for porter, I proposed we should try its effect: this was consented to, and I held a pint of porter to his lips; he drank it off at a draught—soon fell into a sound sleep; when he awoke he was perfectly free from delirium, and from that moment his recovery rapidly progressed.

In relating this case, gentlemen, I do not mean to inculcate the propriety of the invariable use of stimulus, but I do believe that in most cases it will be found a safer remedy than bleeding, more particularly in London, or any crowded city; nor have I formed this judgment from the solitary case just mentioned, but it is an opinion founded upon my own experience and the practice of my colleagues in this hospital as well as in private.

A lady applied to an eminent surgeon, to ascertain from him whether a small encysted tumor could be removed with perfect safety from her head; to which he replied, "certainly." The operation was immediately performed, but seven days afterwards she was dead from an attack of erysipelas.

The next case, as the patient was not attacked by erysipelas after the operation, may be considered out of place with regard to our present considerations; I have mentioned it, however, merely to exemplify the necessity of ascertaining the real constitutional condition before you venture to submit a patient to any mechanical lesion.

A short time ago, an individual came under my care with an external pile and a fissure in the mucous membrane of the rectum ; he was considerably out of health, and attributed all his ailments to the sufferings he experienced in the passing of his motions, owing to the local disease ; he urged me to relieve him by operation. I kept him, however, a week or ten days under my care before I operated, and by soothing remedies had somewhat improved his condition, when I removed the external pile, and drew the bistoury across the fissure, the whole time of the operation not exceeding half a minute. The patient felt immediate relief after the operation ; he had little or no pain in the passage of his motions, but in the course of four or five days he was seized with symptoms of subacute peritonitis ; calomel, and opium, and leeches were ordered, but four days afterwards he died.

Upon examination of the body, he was found to be the subject of granular kidneys, (the morbus Brightii) which no doubt had caused his death.

It had been ascertained, during life, by my dresser, that his urine was albuminous ; but I considered the severity of his suffering demanded the performance of this slight operation ; although the sequel renders it a matter for consideration whether I was right, under these circumstances, in subjecting him to a fresh source of irritation.

From such cases as these you must be impressed, gentlemen, with the necessity of doing everything which the science of surgery can insure, so far as lies in your power, to place your patient in the greatest state of security before you subject him to any surgical operation, and even then never promise that any operation, however simple, will be perfectly free from danger ; for depend upon it, it is as unwise to treat slightly the most trifling incisions of the skin, as it is dishonest to attach to an operation more importance than it justly deserves.

Some surgeons suppose that it is better to perform what are usually considered simple operations at the moment, than to allow the dread of anticipation to remain in the mind of the patient, and then proceed to act upon this opinion without any preliminary precaution. There are, however, I believe, but few patients who will not duly appreciate the cautious recommendation of a surgeon to submit to some little preparatory discipline, and he will gain much more confidence from the patient by this display of his judgment, than from the hasty recklessness which evinces boldness and self seliance, rather than judicious precaution.—*Brit. Amer. Jour. of Med. and Phys. Science.*

